# Clinical research advances of isavuconazole in the treatment of invasive fungal diseases

**DOI:** 10.3389/fcimb.2022.1049959

**Published:** 2022-12-01

**Authors:** Tingting Zhang, Yuyan Shen, Sizhou Feng

**Affiliations:** ^1^ State Key Laboratory of Experimental Hematology, National Clinical Research Center for Blood Diseases, Haihe Laboratory of Cell Ecosystem, Institute of Hematology and Blood Diseases Hospital, Chinese Academy of Medical Sciences and Peking Union Medical College, Tianjin, China; ^2^ Hematopoietic Stem Cell Transplantation Center, Tianjin Institutes of Health Science, Tianjin, China

**Keywords:** isavuconazole, invasive fungal diseases (IFD), invasive aspergillosis, invasive mucormycosis, invasive candidiasis

## Abstract

**Purpose:**

Invasive fungal diseases (IFD) are a major global public health concern. The incidence of IFD has increased the demand for antifungal agents. Isavuconazole (ISA) is a new triazole antifungal agent that has shown promising efficacy in the prophylaxis and treatment of invasive fungal diseases. The aim of this review is to summarize the recent real-world experiences of using ISA for the treatment and prevention of IFD.

**Methods:**

We performed a comprehensive literature search of the MEDLINE, PubMed, Embase, and Cochrane databases for clinical applications of ISA in the real world. Tables and reference lists are presented for this systematic review.

**Results:**

IFD poses a major threat to public health and causes high mortality rates. ISA may provide a good treatment. For example, the efficacy of ISA in the treatment of invasive aspergillosis (IA) is comparable to that of voriconazole, and its efficacy in the treatment of invasive mucormycosis (IM) is similar to that of liposomal amphotericin B (L-AmB); therefore, ISA is recommended as the first-line treatment for IA and IM. ISA can also achieve good efficacy in the treatment of invasive candidiasis (IC) and can be used as an alternative to de-escalation therapy after first-line drug therapy. In addition, most studies have shown the efficacy and safety of ISA for the prophylaxis of IFD.

**Conclusion:**

Taken together, ISA are expected to become a new choice for the treatment and prevention of IFD because of their good tolerability, high bioavailability, and few drug interactions.

## Introduction

Isavuconazole (ISA) is a novel triazole antifungal agent approved in 2015 for the treatment of invasive aspergillosis (IA) and invasive mucormycosis (IM) (Ananda-Rajah and Kontoyiannis, 2015). ISA is available in oral and intravenous dosage forms and has the advantages of high bioavailability, long *in vivo* half-life, broad antifungal spectrum, and high safety. Studies have confirmed that oral ISA can be used as a replacement for posaconazole (POS) to prevent invasive fungal disease (IFD) in patients with high-risk hematological diseases or allogeneic hematopoietic stem cell transplantation (allo-HSCT) (Van Matre et al., 2019). In addition, ISA oral medication or intravenous sequential oral administration has comparable efficacy to voriconazole (VOR) in the treatment of IA. Similarly, its efficacy in the treatment of IM is comparable to that of liposomal Aphotericin B (L-AmB) (Jenks et al., 2019). Moreover, for the treatment of invasive candidiasis (IC), especially rare IFD and IFD involving the central nervous system (CNS), ISA has also achieved good curative effects (Cornely et al., 2018; Perrone et al., 2020). This article reviews the clinical application of ISA in the treatment and prevention of IFD, aiming to provide new insights for the treatment of IFD.

## Pharmacokinetics and pharmacodynamics

**Figure 1:**
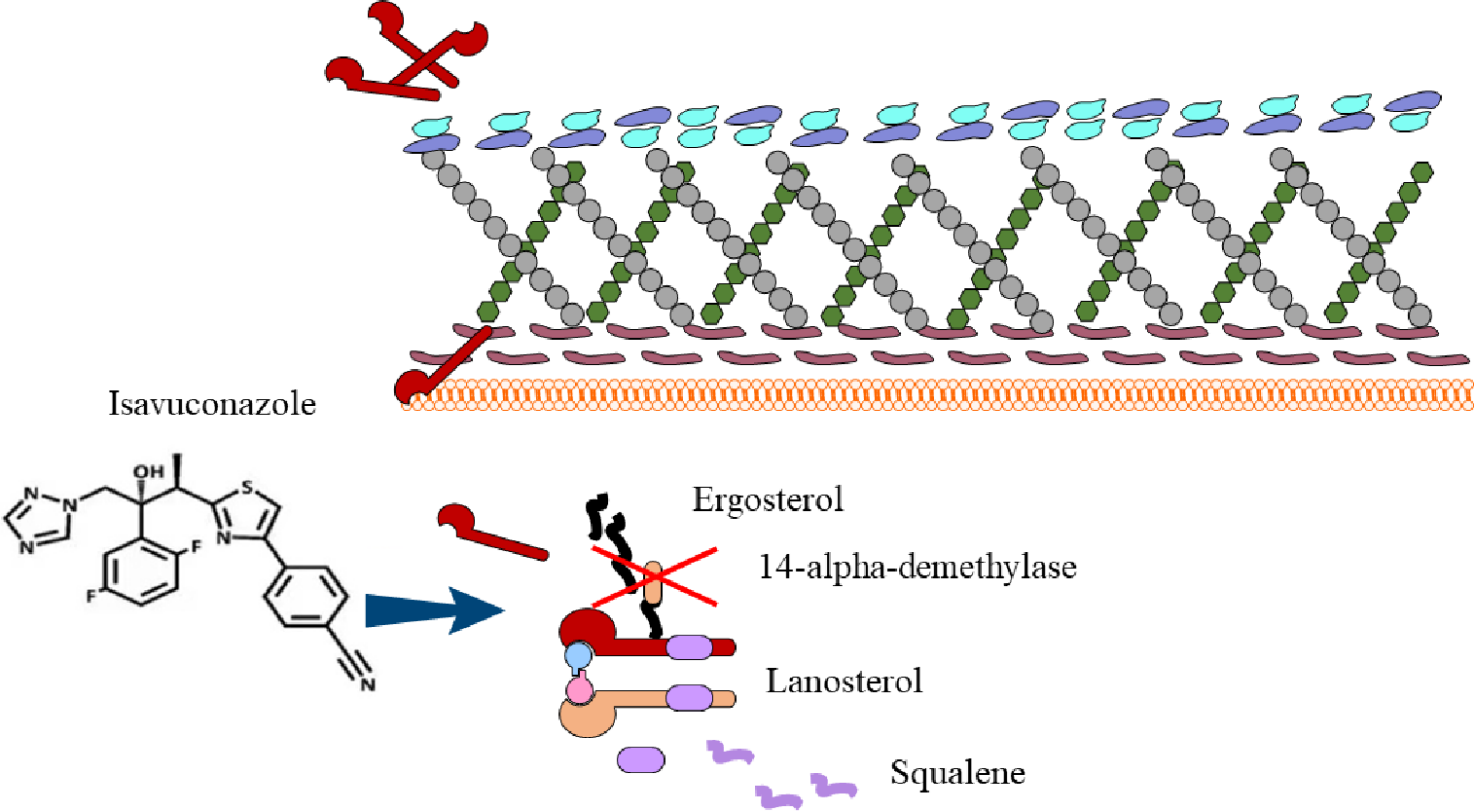
The chemical structure of ISA and its antifungal mechanism.

ISA can inhibit the cytochrome lanosterol 14α-demethylase (CYP51), which destroys the structure and function of the fungal cell membrane by blocking the synthesis of ergosterol on the fungal cell membrane (Shirley and Scott, 2016). The chemical structure of ISA and its antifungal mechanism are showed in **Figure 1**. The side chain in the ISA molecule has a high affinity for the fungal CYP51 protein, which endows it with a broad antifungal spectrum and efficacy against fungi resistant to other triazole antifungals. In terms of antifungal activity, ISA has strong activity against clinically common yeasts and molds, including *Cryptococcus* [minimum inhibitory concentration (MIC) ≤ 0.5ug/ml], *Rhodotorula* (MIC:0.03–0.125 μg/ml), *Trichospora* (MIC:0.002–0.5 µg/mL), *Histoplasma* (MIC:0.125–2 µg/mL), and most *Candida* (MIC <2 µg/mL) (Pettit and Carver, 2015). ISA also has a curative effect against some strains resistant to L-AmB, caspofungin, itraconazole, and emerging pathogens such as *Aspergillus lentus* (Datta et al., 2013).

Due to its good water solubility, the bioavailability of ISA oral administration can reach 98%, and its absorption is not affected by food and gastric acid pH value (Ledoux et al., 2018). For patients with severe conditions requiring nasoenteric tubes who cannot take ISA orally, 83.3% can still achieve effective therapeutic concentrations by opening ISA capsules and administering them enterally (Dieringer et al., 2022). Similarly, McCreary EK (McCreary et al., 2020) revealed that by opening the ISA capsule and administering it enterally, plasma concentrations similar to those of intravenous administration could be achieved. For adults, it is currently recommended to administer 200 mg every 8 h for the first 2 days and then change to 200 mg once daily for maintenance (Ledoux et al., 2018). Currently, there is no uniform standard for the dosage of ISA in children (Naeem et al., 2021). Decembrino N (Decembrino et al., 2020) et al. reported that for adolescents aged 2-17 years, a dose of 10 mg/kg every 8 h for the first 2 days, and then once a day, can obtain safe and effective plasma concentrations. In children aged 6 months to 1 year, the dose was reduced to 6 mg/kg.

The protein binding rate of ISA is as high as 99%, mainly binding to albumin in tissues, and the apparent volume of distribution of the drug can reach 300–500 L, indicating that ISA have good tissue permeability and can enter the CNS through the blood-brain barrier. In animal models, changes in drug concentrations in different mouse tissues or organs were detected 1 h after administration. The results showed that the drug concentrations were highest in bile, liver, adrenal, and brown adipose tissues, while the lowest drug concentrations were in bones and lenses of eyes (Schmitt-Hoffmann et al., 2017). Notably, in animal models, the average drug concentration in mouse brain tissue is 1.8 times higher than that in serum (Schmitt-Hoffmann et al., 2017; Ledoux et al., 2018), suggesting that ISA can pass through the blood-brain barrier and reach effective concentrations in the brain parenchyma. In addition, the maximal plasma concentration (Cmax) achievable 2-3 hours after oral administration of 200 mg of ISA in healthy volunteers was 2.59 ± 0.449 μg/mL (Schmitt-Hoffmann et al., 2016). After intravenous injection of the same dose of ISA, Cmax was 2.47 ± 0.374 μg/mL, which was not significantly different from oral administration (Townsend et al., 2018). Therefore, both oral and intravenous administrations are suitable for clinical applications. In addition, ISA requires less monitoring of blood concentration than other antimicrobials (Kably et al., 2022). The pharmacokinetic characteristics of ISA and other major antifungal drugs are summarized in **Table 1**.

**Table 1:** Comparison of ISA with other antifungal drugs.

	ISA	VOR (Malani et al., 2015; Jin et al., 2016)	L-AMB (Stone et al., 2016; Groll et al., 2019)	POS (Chen et al., 2020; Van Daele et al., 2020)
**Dosage form**	Oral and intravenous	Oral and intravenous	Intravenous	Oral (tablet and suspension) and intravenous
**Oral bioavailability**	Comparable to intravenous administration	Lower than intravenous administration	No oral dosage form	Lower than intravenous administration
**Food Effects**	None	Decreased Absorption	None	Oral Suspension Increases Absorption
**Pharmacokinetics**	Linear and predictable	Nonlinear	Nonlinear	Nonlinear
**Between-patient variation**	Small	Significant	Significant	Significant
**Solubility Requirements**	None	Cyclodextrin for intravenous formulations	None	Cyclodextrin for intravenous formulations
**CYP450 Effect**	Slight	High	None	Moderate
**Adverse events**	Well tolerated	Skin, ocular, liver and kidney	Nephrotoxicity	Well tolerated
**Drug monitoring**	Unnecessary	Necessary	Unnecessary	Suspension required
**Patients with renal insufficiency**	Applicable	Intravenous preparations are not suitable	Long-term use requires caution	Intravenous preparations are not suitable
**Patients with hepatic insufficiency**	Use with caution in severe hepatic impairment	Caution recommended; dose adjustment required for severe hepatic impairment	No recommendation data	Use with caution in severe hepatic impairment

## Drug-drug interaction

ISA is a substrate for cytochrome CYP3A4; therefore, inhibitors or inducers of this enzyme can alter ISA concentrations in the body. Potent inducers of CYP3A4, such as rifampicin, carbamazepine, and long-acting barbiturates, significantly reduce the plasma concentration of ISA and are therefore not recommended in combination with ISA (Administration, 2015). ISA is a moderate inhibitor of CYP3A4, inhibiting the metabolism of sirolimus, tacrolimus, and cyclosporine to varying degrees, resulting in increased concentrations of these agents in the body. Therefore, when using such drugs, it is necessary to monitor the drug concentration and reduce the dose appropriately (Schwarz et al., 2019). ISA has little effect on other CYP3A4 substrates such as midazolam and atorvastatin, and no dose adjustment is necessary (Stott and Hope, 2017). ISA had little effect on cytochrome CYP2C9 and CYP2C19, and no dose adjustment was required when co-administered with warfarin and omeprazole. ISA is a mild inhibitor of the drug transporter P-glycoprotein (P-gp), which can increase serum digoxin (P-gp substrate) levels. Thus, the dose of digoxin should be reduced when co-administered with ISA (McCarthy et al., 2018).

## Clinical efficacy

### IA

*Aspergillus fumigatus* is the most common IA, and up to three million people worldwide are infected each year (Barac et al., 2019). The CAESAR study showed that *Aspergillus* was the most common pathogen in probable or proven IFD patients after allo-HSCT, accounting for approximately 70.6%, and its mortality rate was as high as 20-25% (Sun et al., 2015). Atypical clinical manifestations may only express symptoms, such as fever and hemoptysis. Once *Aspergillus* invades pulmonary blood vessels, chest CT examination may show dense nodules, as well as typical “crescent sign,” “halo sign” cavity formation, and other imaging features. If *Aspergillus* invades the alveoli or bronchiolar walls, chest CT examination may show small nodular shadows, ground glass-like changes, tree-in-bud signs, and other changes (Russo et al., 2020). VOR has long been the cornerstone of IA treatment, but its clinical application is limited owing to its high incidence of adverse effects. In recent years, after the launch of ISA which combines comparable curative effects and better safety, ISA has also been recommended as the first-line treatment for IA (Denning et al., 2016).

Studies have shown that ISA has a significant effect on the treatment of IA (Rybak et al., 2015). Maertens et al. (2016) conducted a phase III, double-blind, global, multicenter, randomized controlled noninferiority clinical trial (SECURE trial) to compare the efficacy of ISA and VOR in the treatment of IA. Their study included 527 patients diagnosed with proven, probable, or possible IA or other filamentous fungi. The patients were treated with ISA or VOR at a 1:1 matching ratio. Patients in the ISA group received intravenous infusion of ISA 200 mg three times a day for the first two days, and then changed to daily intravenous infusion or oral ISA 200 mg once a day from the third day. In contrast, patients in the VOR group received 6 mg/kg intravenous infusion twice on day 1, 4 mg/kg twice intravenous infusion on day 2, changed to 4 mg/kg daily intravenous infusion from day 3, or oral VOR tablets 200 mg twice daily. The results showed that the 42-day all-cause mortality in the ISA and VOR groups was 19% and 20%, respectively (95% confidence interval (CI) -7.8-5.7). Because the upper limit of the 95% CI did not exceed the 10% pre-specified value for noninferiority, there was no significant difference in mortality between the two groups. Likewise, there was no significant difference in response rates (including complete and partial responses) between the two groups at the end of treatment (35% and 36.4%, respectively). In addition, the incidence of adverse effects in the ISA group was 42% and 60% in the VOR group (P<0.001). The incidence of eye (15% vs. 27%, P=0.002), skin and subcutaneous tissue disease (33% vs. 42%, P=0.037), hepatobiliary system (9% vs. 16%, P=0.016)) and other adverse events in the ISA group was significantly lower than that in the VOR group, respectively. Among patients treated with ISA, 14% discontinued treatment due to adverse events, compared with 23% in the VOR group (Kontoyiannis et al., 2014). Therefore, the SECURE study confirmed that the efficacy of ISA in the treatment of IA is comparable to that of VOR, with a lower incidence of adverse effects.

In addition, Horn et al. (2016) conducted a subgroup analysis of the SECURE study and showed that ISA could significantly decrease the length of hospital stay in patients with moderate to severe renal impairment. The average length of hospital stay was 9 days in the ISA group, compared with 19 days in the VOR group. Therefore, ISA is also an effective therapeutic agent for patients with impaired renal function. Similarly, Townsend et al. (2017) conducted a phase I clinical trial to further evaluate the effect of renal impairment on the pharmacokinetics of ISA. Compared with healthy subjects, the maximum plasma concentration of ISA in patients with mild renal impairment increased by 4%, while the maximum plasma concentration of ISA in patients with moderate renal impairment, severe renal impairment, and end-stage renal disease was 7%, 4%, and 21% lower than that in normal subjects, respectively. This study showed that there were no significant differences in ISA pharmacokinetics between patients with mild, moderate, or severe renal impairment and healthy subjects. Therefore, ISA dose adjustment was not required in patients with renal impairment.

Real-world studies have shown a satisfactory effect of ISA in the treatment of IA among patients with hematological diseases or solid organ transplantation. Dagher et al. (2022) summarized 200 hematological malignancies or allo-HSCT cases with IFD at the MD Anderson Cancer Center between April 1, 2016, and January 31, 2020, including proven (11 cases), probable (63 cases), and possible (126 cases). *Aspergillus* was the main pathogen. A favorable response rate with ISA was observed in 40% of patients at 6 weeks and 60% at 12 weeks. In this study, ISA was used as monotherapy in 30% of the cases and as combination therapy in 70% of the patients. There was no significant difference in efficacy between ISA monotherapy and combination therapy (P = 0.16, 6 weeks; P = 0.06, 12 weeks). Therefore, the ISA combination therapy did not demonstrate an advantage over ISA monotherapy. Cattaneo et al. (2019) conducted a multicenter retrospective study of 122 patients with hematological diseases who had possible (51 cases), probable (59 cases), or proven (12 cases) IFD in 17 medical centers in Italy. The median age was 57.5 (19-80) years, with hematological malignancies in 113 patients, aplastic anemia in 3, neutropenia in 97, and allo-HSCT in 41. IA accounted for up to 72.7% of patients in this study. The results showed that the overall response rate of ISA in the treatment of IFD was 67.2%, with a complete response rate of 51%. The response rate was 93% in 71 patients with probable or proven IA. In this study, ISA was used as first-line treatment in 35% of patients and second-line treatment in 65% of patients. ISA treatment was effective in both groups, with response rates of 60.5% and 70.9%, respectively (P=0.24). The one-year overall survival was 49.9%, of which the one-year overall survival was 68% for those who responded to ISA treatment and only 14.1% for those who did not respond to ISA treatment (P<0.0001). In addition, Monforte et al. (2022) reported the efficacy of ISA in the treatment of 53 solid organ transplant IFD patients, of which 43 (81.8%) had IA. After a median of 81 days of ISA treatment, 50.9% of patients showed clinical cure, and 34% of patients developed mild transaminase elevation. Of note, seven patients received ISA combined with mTOR inhibitors with good tolerance. Therefore, for the treatment of IA in patients with hematological diseases and solid organ transplantation, ISA was a worthy first-line treatment agent.

ISA can also be safely and effectively administered to patients with intolerance or resistance to other triazoles. In a retrospective study of the long-term treatment of patients with chronic pulmonary aspergillosis (CPA), 20 patients were treated with ISA and 21 patients were treated with VOR. The incidence of adverse events was significantly lower in the ISA group than in the VOR group (60% vs. 86%, P=0.02). Five patients in the ISA group were previously intolerant to other triazole antifungals but tolerated standard doses of ISA (Bongomin et al., 2019). Similarly, Nwankwo et al. (2022) shared the experience of ISA as salvage therapy for CPA patients from 2016 to 2021; 132 patients who were resistant to other triazoles were treated with ISA as salvage therapy. The blood concentration was higher than 1 mg/L among 94.8% of the patients with ISA, and 72% of the patients reached the standard dose of ISA blood concentration (> 2 mg/L). Although 61.8% of the patients were previously unable to tolerate other azole antifungals due to toxicity, 68% of the patients in this study tolerated ISA well.

De Leonardis et al. (2020) reported a case of a 3-year-old child with acute lymphoblastic leukemia who developed concurrent IA infection of the lung and brain during induction therapy. The child belonged to the type of VOR CYP2C19 fast metabolizer. After failure of first line VOR treatment, the patient was converted to ISA treatment. The child had no obvious adverse effects, and the brain infection foci shrank. Similarly, Assaf et al. (2020) reported a patient with invasive *Aspergillus fumigatosis* and sternal osteomyelitis after heart transplantation who discontinued VOR due to severe cutaneous and neurotoxicity and subsequently improved after switching to ISA monotherapy. Therefore, patients intolerant or resistant to other triazole antifungal drugs should also consider switching to ISA therapy.

Using economic modeling, many experts in the United States, United Kingdom, and Sweden have conducted cost-effectiveness studies of ISA for the treatment of IA and found that ISA were ideal agents with optimized cost-effectiveness for the treatment of IA (Floros et al., 2019; Jeck et al., 2021). Harrington et al. (2017) employed an economic model to assess the cost-effectiveness of ISA versus VOR in hospitalized IA patients and included data from the SECURE study for further validation. The results showed that, compared with VOR, ISA could save costs for hospitalized patients, including medication, adverse event costs, and readmissions. Therefore, the 6th European Leukemia Infection Conference and the European Society of Clinical Microbiology and Infectious Diseases recommended that ISA, like VOR, could be used as a first-line treatment for IA patients with hematological malignancies or allo-HSCT (Tissot et al., 2017; Ullmann et al., 2018). In recent years, with the increase in IA related to the novel coronavirus 19 (COVID-19), the European Confederation of Medical Mycology (ECMM) and the International Society of Human and Animal Mycology (ISHAM) jointly issued guidelines recommending ISA as first-line therapy for COVID-19 related pulmonary IA (Koehler et al., 2021).

### IM

IM is the third most common IFD in allo-HSCT patients, and the 1-year overall survival rate in IM patients after HSCT is as low as 28% (Bao et al., 2022). Because of the difficulty in obtaining deep tissue samples in many cases and the low sensitivity of diagnostic methods, the incidence of IM in the real world may be higher (Skiada et al., 2020). In IM patients, if treatment is delayed for 6 days after diagnosis, the 12-week mortality rate will approximately double to 82.9% (Chamilos et al., 2008). The most common manifestation is nasal-orbital-brain infection, usually with symptoms similar to acute sinusitis, such as fever, headache, sinus pain, or nasal congestion (Reid et al., 2020). Patients with IM with hematological malignancies often manifest either pulmonary IM or disseminated type. Imaging of patients with pulmonary IM shows a characteristic “reverse halo sign,” which is more common in patients with neutropenia (Hammer et al., 2018; Marchiori et al., 2018). Current guidelines mainly recommend L-AmB as an agent for the initial treatment of patients with IM. Although L-AmB reduces the disadvantage of AmB nephrotoxicity, its practical application is difficult because the recommended dose for IM treatment needs to be increased to 5-10 mg/kg (Jestin et al., 2021). Furthermore, despite its anti-mucor activity, POS has not been approved for the treatment of IM (Brunet and Rammaert, 2020). In economic modeling, Bagshaw et al. (2017) found that, compared with the standard treatments for IM, ISA could significantly reduce the cost of drugs and readmissions. Currently, ISA has been approved by the FDA for the treatment of IM, and the guidelines recommend that based on surgical debridement, ISA is the most suitable drug for the initial treatment of patients with impaired renal function (Cornely et al., 2019).

Marty et al. (2016) conducted an open-label multicenter study, including 37 proven and probable IM patients from 34 centers around the world, to evaluate the efficacy and safety of ISA in the treatment of IM, and compared its efficacy with that of an AmB-based control group in a paired case-control study. After 42 days of treatment, 11% of the patients had a partial response, 43% had stable IFD, 3% had progressive IM, and 35% had died. The 84-day overall survival rates in the ISA and AmB groups were 57% and 50%, respectively (P=0.653), while the all-cause mortality rates on day 42 were 33.3% and 41.3%, respectively, with no statistical difference (P=0.595). In this study, the incidence of adverse events in the ISA group was similar to that in the SECURE trial and no serious organ damage was detected. Therefore, ISA could be considered a promising approach in the treatment of IM with a safety profile and similar curative effects to AmB-based therapy. In addition, Thompson et al. (2022) conducted a multicenter, non-interventional registration study in which 42 patients with IM were initially treated with ISA. Among the patients who received ISA monotherapy or ISA combined with other antifungal agents, the day-42 all-cause mortality rates were 33.3% and 41.3%, respectively, indicating that ISA single-agent initial treatment for IM achieved good curative results.

Miller et al. (2021) compared the efficacy of 28 cases of L-AmB monotherapy and 21 cases of L-AmB combined with POS or ISA in the treatment of IM after allo-HSCT. The failure rates between the two groups were 64% and 43%, respectively (P =0.136). Among the patients who initially received L-AmB monotherapy, 10% switched to L-AmB plus ISA after treatment failure. The 30-day mortality rates in the L-AmB monotherapy and the L-AmB combined POS/ISA treatment groups were 43% and 28%, respectively. In the combination group, 33.3% of the patients improved without adverse events compared with 10.7% in the L-AmB monotherapy group. The results of this study preliminarily confirmed that the combined treatment of ISA or POS with L-AmB is safer and more effective than L-AmB monotherapy. Based on the above research results, the 2019 guidelines issued by the European Federation of Medical Fungi emphasized that for suspected or confirmed IM, based on debridement or previous renal impairment, ISA or POS intravenous administration was strongly recommended. For patients whose disease progressed at the time of response assessment after initial treatment with L-AmB, switching to ISA or POS for salvage therapy is also strongly recommended (Cornely et al., 2019).

It is worth noting that ISA treatment of children with IM has also initially shown effectiveness, especially as a rescue therapy for children when other agents are ineffective or intolerable. Muggeo et al. (2019) reported on 11 children with IM who underwent surgical debridement combined with L-AmB treatment. Among the children who received POS maintenance therapy, six achieved complete remission, while two who received ISA single-agent maintenance therapy achieved complete remission. Furthermore, Ashkenazi-Hoffnung et al. (2020) analyzed the efficacy of ISA rescue therapy in 4 children with IM from 2015 to 2019. All four children achieved remission after ISA monotherapy or in combination with other agents. Barg et al. (2018) reported two cases of rhino-orbital-cerebral IM in children with leukemia. The infections progressed after L-AmB monotreatment, but were completely relieved after switching to L-AmB combined with ISA salvage therapy. Similarly, Pomorska et al. (2019) reported the case of a 7-year-old child with acute lymphoblastic leukemia who developed disseminated IM during chemotherapy. The infection progressed after L-AmB combined with caspofungin, and complete remission was achieved after ISA administration. Cornu et al. (2018) reported the case of a 3-year-old child with acute lymphoblastic leukemia who developed disseminated IM. After switching from VOR to ISA monotherapy owing to renal damage, the patient was successfully treated. Alali et al. (2022) reported the case of a child with CNS-relapsed acute lymphoblastic leukemia who developed solitary cerebral IM. After debridement and L-AmB administration, the infection spread, but the infection resolved after switching to oral ISA single-agent rescue therapy. The above studies proved that ISA is also a safe and effective option for children with IM. Most of the current studies showed that a daily therapeutic dose of 100 mg was a safe and effective regimen for children with IM weighing less than 30 kg. The first ISA blood concentration monitoring can be performed on the 10th day of ISA treatment and every 2 weeks thereafter (Zimmermann et al., 2022). Similarly, Arrieta et al. (2021) evaluated the effects of ISA on the pharmacokinetics, safety and tolerability of 46 immunocompromised children, and the results showed that ISA was well tolerated at 10 mg/kg intravenously or orally, children could achieve plasma concentrations similar to adults. In view of the existing research, ISA initially exhibited the advantages of being safe and effective in the treatment of children with IM, but more large-scale clinical trials are still needed for further support.

### IC

IC mostly occurs in immunocompromised patients, and studies have shown that the mortality rate of IC in patients with hematological diseases is as high as 40%-60% (Ricotta et al., 2020). *Candida* spp. can form diffuse small abscesses, infected emboli, and hemorrhagic infarcts through hematogenous dissemination. On chest CTs, IC is often manifested as disseminated miliary nodules with diameters less than 1 cm (Gonzalez-Lara and Ostrosky-Zeichner, 2020). ISA also has curative effects in the treatment of IC. In a double-blind, randomized phase III clinical trial, the ACTIVE study compared the efficacy of ISA and caspofungin in the treatment of IC (Kullberg et al., 2019). Patients were randomly divided into an ISA treatment group (n=221) and a caspofungin-treated group (n=219). After 10 days of intravenous administration, the patients in the two groups were sequentially treated with oral drugs. Patients in the ISA group were treated with ISA, while those in the caspofungin group were treated with VOR. The results showed that, after the end of intravenous preparation, the median time of negative blood culture was 4 days in the ISA group and 3 days in the caspofungin group (P=0.59). The overall response rates were 60.3% and 71.1%, respectively (adjusted difference between groups: -10.8%; 95% CI -19.9-1.8). Because the lower bound of the 95% CI in this result (19.9%) was lower than the prespecified noninferiority value (15%), the study did not show noninferiority of ISA for IC. Two weeks after the end of all treatments, the overall response rates of patients in the ISA and caspofungin groups were similar at 54.8% and 57.2%, respectively (adjusted difference between groups, -2.7%; 95% CI -12.2-6.8). In this study, ISA did not show the expected efficacy advantage; however, there was no significant difference in all-cause mortality, safety, and blood Candida clearance between the two groups on days 14 and 56. Further, 34.7% of the patients in the ISA group and 39.8% of the patients in the caspofungin group completed the transition from intravenous to oral antifungal therapy, with an overall response rates for the two groups of patients who switched to oral preparations of 82.6% and 77.5%, respectively. The above results demonstrate that ISA might not be as effective as caspofungin in the treatment of IC, but it remains an alternative for sequential medication after IC control.

Another phase II clinical trial compared the efficacy and safety of three oral ISA and fluconazole regimens in the treatment of esophageal candidiasis (Viljoen et al., 2015). The specific groups were as follows: Group A: ISA 200 mg on the first day, then 50 mg once daily; Group B: ISA 400 mg on the first day, then 400 mg once a week; Group C: ISA 400 mg on the first day, then 100 mg once daily; and Group D: fluconazole 200 mg on the first day, then 100 mg once a day. The minimum treatment duration for each group of patients was 14 days, and 153 patients were evaluated for efficacy at the end of the treatment. In this study, 146 patients (95.4%) achieved endoscopically confirmed clinical remission. The efficacy of the three ISA treatment regimens was similar to fluconazole. Also, the incidence of adverse events was also similar in groups A (n=22), B (n=18), and D (n=22) at 55%, 45%, and 58%, respectively; however, in group C (n=29) they were 71%. Based on the above findings, ISA 50 mg once daily or 400 mg once weekly and fluconazole 100 mg once daily showed no significant differences in efficacy and safety for treating esophageal candidiasis. Moreover, Odysseos et al. (2022) reported a patient with IC after liver transplantation who was cured and discharged after treatment with ISA combined with L-AmB. The patient tolerated the combination therapy well and no adverse effects were observed. At present, echinocandins are still the first-line treatment options for IC, and ISA can be used as an alternative for de-escalation after first-line treatment in patients with IC.

### Other IFD

The relevant data of ISA for the treatment of other rare IFDs are scarce, but still reveal good curative effects. Thompson et al. (2016) analyzed 38 patients with rare fungal diseases (including nine patients with cryptococcosis, ten patients with *Paracoccidioides* spp., nine patients with *Coccidioides* spp., seven patients with *Histoplasma* spp., and three patients with *Blastomyces* spp.) who received ISA treatment in the VITAL study. By the end of treatment, the overall response rate was 63%, and 21% of patients had no disease progression. Together, these data tentatively suggest that ISA remains a good option for the treatment of rare IFD.

ISA has good blood-brain barrier penetration and a therapeutic effect on intracranial fungal infections. Schwartz et al. (2020) observed 36 patients with IFD involving the CNS, including 11 with mucormycosis, 8 with aspergillosis, and 5 with cryptococcosis. By the end of treatment, 58.3% of the patients achieved complete or partial clinical remission. Naeem et al. (2021) reported a pediatric case of refractory coccidioides meningitis that relapsed after receiving VOR and L-AmB treatment and discontinued treatment due to severe nephrotoxicity and neurotoxicity. After daily administration of 100 mg of ISA, the child improved rapidly and was discharged from the hospital. After 21 months of follow-up, the child’s condition was stable, and no recurrence occurred. Taken together, ISA have great therapeutic potential for the effective treatment of IFD and IFD involving the CNS.

### Prospect of ISA in the prevention of IFD

Numerous studies have demonstrated that ISA also have therapeutic potential for IFD prophylaxis with fewer adverse effects than other antifungal treatments (Hassouna et al., 2019; Samanta et al., 2021). Details are presented in **Table 2**. Samanta et al. (2021) retrospectively analyzed the differences in the efficacy of ISA or VOR in the prevention of IFD in lung transplant patients. The median prevention time in ISA and VOR groups were 3.4 months and 3.1 months, respectively. After one year, the incidence of IFD in both groups was 8%, with no significant difference. The incidence of drug discontinuation owing to side effects was much lower in the ISA group than in the VOR group (11% vs. 36%, P=0.0001). Bose et al. (2021) studied the efficacy of ISA in the prevention of IFD in patients with hematological malignancies. During the 30-day follow-up period of 65 patients, four patients (6%) had proven or probable IFD, and eight patients (12%) had possible IFD. All patients in this study had ISA plasma concentrations >1 µg/mL and were generally well tolerated, with only three patients (5%) experienced mild to moderate transaminase elevations, and there were no changes in the QT intervals. Thus, ISA are effective and safe in preventing IFD in patients with hematological diseases.

**Table 2:** Summary of ISA prophylactic antifungal treatments.

Author	Test design	Patient type	Number of people in the ISA prevention group	Control group agent and number of people	Curative effect	Survival outcomes	Adverse events	Attitudes towards ISA preventive use
Hassouna et al. (2019)	retrospective		17	N/A	No patient developed bIFIs.	The 6-week mortality rate was 24%.	N/A	Supportive
Bowen et al. (2019)	single center, retrospective	AML patients	98	N/A	bIFIs:8.2%	bIFIs-related mortality: 2.04%.	Incidence of discontinuation due to side effects: 4.3%	Supportive
Stern et al. (2020)	Single-center, prospective	Allo-HSCT patients	95	N/A	bIFIs: 3.1%, with a cumulative incidence of prevention failures of 10.7%	bIFIs-related mortality: 3.2%.	The incidence of discontinuation due to adverse reactions: 7.4%. Incidence of abnormal liver function: 5.3%	Supportive
Bose et al. (2021)	Prospective, open-label, phase 2 clinical trial	Treatment-naive AML or MDS patients	65	N/A	Confirmed and clinically diagnosed IFD (6%), probable IFD (12%)	42-day bIFIs-related mortality (3.08%)	Mild to moderate liver function abnormalities (5%), no patients with QT prolongation	Supportive
Samanta et al. (2021)	single center, retrospective	lung transplant patients	144	VOR(156)	bIFI:ISA(7%), VOR(8%), P=1.0	bIFIs-related mortality:ISA(1.39%), VOR(1.28%), P>0.05	Incidence of discontinuation due to adverse reactions:ISA(11%), VOR(36%), P = 0.0001.	Supportive
Bogler et al. (2021)	prospective	Allo-HSCT patients	95	VOR(210)	bIFIs:ISA(3.2), VOR(2.8%), P=0.081	all-cause mortality:ISA(6.3%), VOR(2.4%), P=0.089	Incidence of discontinuation due to hepatotoxicity:ISA(5.26%), VOR(22.8%), P= 0.0002	Supportive
Vu et al. (2021)	single center, retrospective	Hematological patients and HSCT patients	26, Primary Prevention (21), Secondary Prevention (5)	N/A	Primary prevention bIFIs: 11.2%	bIFIs	N/A	Supportive
Fontana et al. (2020)	single center, retrospective	Hematological patients and allo-HSCT patients	85	POS(68)VOR(88)	bIFIs:ISA(10.2%), POS(4.1%), VOR(1.1%), P=0.2	related:ISA(8.24%), POS(N/A), VOR(N/A)	N/A	Opposing

ISA, isavuconazole; N/A, not available; POS, posaconazole; VOR, voriconazole. bIFI, breakthrough invasive fungal infection; AML, acute myeloid leukemia; allo-HSCT, allogeneic hematopoietic stem cell transplantation.

Moreover, Stern et al. (2020) initiated a single-arm prospective study to evaluate the prophylactic effect of ISA in 95 allo-HSCT patients. After a median prophylaxis of 90 days, 85% of allo-HSCT patients tolerated long-term use of ISA, with a breakthrough IC incidence rate of 3.1%, and no patient developed IA infection. The results confirmed the feasibility of ISA for prophylactic use in allo-HSCT patients. Similarly, a prospective study by Bogler Y (Bogler et al., 2021) compared the efficacy of VOR and ISA in the prevention of IFD in allo-HSCT patients, in which 210 patients were treated with VOR prophylaxis and 95 patients were treated with ISA prophylaxis. The median duration of ISA primary prophylaxis was significantly longer in the ISA group than that in the VOR group (94 vs. 76 days, P < 0.0001). The incidence of breakthrough invasive fungal infections (bIFIs) at 180 days after transplantation was 2.9% and 3.2% in the VOR and ISA groups, respectively (P = 0.881), while the all-cause mortality rates were 2.4% and 6.3%, respectively (P = 0.889). In contrast, the incidence of hepatotoxicity was significantly higher in the VOR group than that in the ISA group (22.8% vs. 5.26%; P=0.0002). In this study, ISA had a lower incidence of hepatotoxicity, whereas bIFIs and all-cause mortality were similar to those of VOR. In short, most studies have revealed that ISA and VOR are equally effective in preventing IFD in the setting of solid organ transplantation, hematological malignancies, and allo-HSCT, but ISA has fewer side effects. In addition, Bowen et al. (2019) reported that patients with ISA prophylaxis achieved an average drug cost saving of $128.25, compared to patients with POS prophylaxis.

In contrast, Fontana et al. (2020) reported that among 145 hematological malignancies and allo-HSCT patients who underwent ISA for primary antifungal prophylaxis between September 1, 2016, and September 30, 2018, the incidence rate of bIFIs in the ISA prophylaxis group was 10.2%, while those in the POS and VOR prevention groups were 4.1% and 1.1%, respectively (P=0.2). The incidence rate of breakthrough invasive pulmonary aspergillosis (bIPA) in the ISA prevention group was 6.8%, while in the POS and VOR prophylaxis groups they were 1.3% and 0%, respectively (P=0.1). Although the incidence of bIFIs in ISA was statistically indistinguishable from POS and VOR, the investigators concluded that ISA is not suitable for primary antifungal prophylaxis in patients with hematological malignancies and allo-HSCT. Considering that the prevention form of POS and VOR in this study was mainly *via* injection, while the prevention form of the ISA group was tablets, route of administration may have played a role. Other studies have shown that the incidence of bIFIs when using VOR tablets can reach 8.5% (Perreault et al., 2019), whereas the incidence rate of bIFIs when using POS suspension for prevention is as high as 13.5% (Li et al., 2020). Thus, we believe that ISA is still safe and effective agent for the prevention of IFD.

## Adverse events

The most common side effects of ISA treatment are gastrointestinal reactions, including nausea, vomiting, and diarrhea, which rarely lead to discontinuation (Dagher et al., 2022; Monforte et al., 2022). A study of healthy adult subjects in Japan found that the side effects of ISA treatment were mild to moderate, and no serious adverse reactions occurred in the subjects (Shirae et al., 2022). Furfaro et al. (2019) found that gastrointestinal reactions occurred in approximately 31.6% of patients after ISA administration. According to the time-dependent ROC curve, a plasma concentration of 4.87 mg/L is the threshold for toxicity (Furfaro et al., 2019). ISA is hepatotoxic and may result in elevated transaminase levels. Therefore, liver function tests should be serially monitored during ISA treatment. In the SECURE trial, the incidence of drug-related adverse events was 42% in patients treated with ISA and as high as 60% in patients treated with VOR (P<0.001). Other studies also found that the incidence of hepatotoxicity in the ISA group was lower than that in the VOR group (9% and 16%, respectively; P=0.016) (Maertens et al., 2016). In a study of 65 patients with hematological diseases who received ISA for primary antifungal prophylaxis, only 3 patients (5%) had mild to moderate liver function abnormalities, and no patients had QT interval prolongation (Bose et al., 2021). Among the 111 IFD patients treated with ISA, 12.6% had mild to moderate adverse effects such as nausea and vomiting, and 2.7% of the patients had abnormal liver function, but no drug-related deaths occurred (Thompson et al., 2022). In addition, DiPippo et al. (2019) retrospectively analyzed the occurrence of adverse events in 23 patients with leukemia after switching from POS to ISA to prevent or treat IFD. Among them, 20 patients switched to ISA treatment due to POS-related hepatotoxicity. High transaminase levels and the liver function returned to normal. Among the patients treated with ISA, no patients experienced grade 3/4 QT interval prolongation, and no one discontinued ISA treatment due to adverse effects. However, ISA resulted in a dose-related shortening of the QT interval compared with other triazoles which are more likely to cause QT prolongation (Keirns et al., 2017). A mean QT interval decrease of 7.4 ± 5.8% in 24 patients was observed in a total of 26 patients treated with ISA (Mellinghoff et al., 2018). The underlying mechanism of ISA-induced QT shortening is unclear, and ISA therapy should be avoided in patients with familial QT shortening. In summary, the adverse events of ISA mainly include gastrointestinal reactions, abnormal liver function, and shortening of the QT interval; it may also interact with drugs metabolized by the CYP450 system. Therefore, attention should be paid to monitoring blood concentration and adjusting the dose of the agent during combination therapy.

## Conclusion

ISA is a new type of triazole antifungal agent that has shown promising efficacy in the clinical prevention and treatment of IFD (Shirley and Scott, 2016). The efficacy of ISA in the treatment of IA is comparable to that of VOR, its safety and tolerability are better than those of VOR and L-AmB (Maertens et al., 2016), and its efficacy in the treatment of IM is similar to that of L-AmB (Marty et al., 2016). Therefore, ISA is the first-line treatment for patients with IA and IM. ISA can also achieve good efficacy in the treatment of IC and can be used as an alternative to de-escalation therapy after first-line drug therapy has achieved efficacy (Ellsworth and Ostrosky-Zeichner, 2020). ISA is also a suitable option for rare IFD and IFD involving the central nervous system. In addition, ISA is an effective approach to prevent IFD. In summary, ISA is expected to become a new choice for the treatment and prevention of IFD because of its high tolerability, high bioavailability, and few drug interactions.

## Author contributions

TZ wrote the review. YS searched comprehensive literature. SF revised the review. All authors contributed to the article and approved the submitted version.

## Funding

This review was supported by Tianjin Municipal Science and Technology Commission Grant (21JCZDJC01170), Haihe Laboratory of Cell Ecosystem Innovation Fund (grant number HH22KYZX0036), Innovation Fund for Medical Sciences (CIFMS) (grant numbers 2021-I2M-1-017 and 2021-I2M-C&T-B-080), and the Youth Program of National Natural Science Foundation of China (No. 81900182).

## Conflict of interest

The authors declare that the research was conducted in the absence of any commercial or financial relationships that could be construed as a potential conflict of interest.

## Publisher’s note

All claims expressed in this article are solely those of the authors and do not necessarily represent those of their affiliated organizations, or those of the publisher, the editors and the reviewers. Any product that may be evaluated in this article, or claim that may be made by its manufacturer, is not guaranteed or endorsed by the publisher.

## Abbreviations

allo-HSCT, allogenic hematopoietic stem cell transplantation; bIFIs, breakthrough invasive fungal infections; CPA, chronic pulmonary aspergillosis; CYP51, cytochrome lanosterol 14α-demethylase; IA, invasive aspergillosis; IC, invasive candidiasis; IFD, invasive fungal disease; IM, invasive mucormycosis; ISA, isovuconazole; L-Amb, liposomal Amphotericin B; POS, Posaconazole; VOR, voriconazole.
